# Author Correction: Intrinsic chiral field as vector potential of the magnetic current in the zig-zag lattice of magnetic dipoles

**DOI:** 10.1038/s41598-023-29687-6

**Published:** 2023-02-15

**Authors:** Paula Mellado, Andrés Concha, Kevin Hofhuis, Ignacio Tapia

**Affiliations:** 1 Facultad de Ingeniería y Ciencias, Universidad Adolfo Ibáñez, Santiago, Chile; 2 Departamento de Física, Facultad de Ciencias, Universidad de Chile, Casilla 653, Santiago, Chile; 3 Laboratory for Mesoscopic Systems, Department of Materials, ETH Zurich, Zurich, Switzerland; 4 Laboratory for Multiscale Materials Experiments, Paul Scherrer Institute, Villigen PSI, Switzerland; 5 Department of Applied Physics, Yale University, New Haven, USA

Correction to: *Scientific Reports* https://doi.org/10.1038/s41598-023-28545-9, published online 23 January 2023

The original version of this Article contained an error in Affiliation 4, which was incorrectly given as:

Laboratory for Multiscale Materials Experiments, Paul Scherrer Institute, Würenlingen, Switzerland.

The correct Affiliation is listed below:

Laboratory for Multiscale Materials Experiments, Paul Scherrer Institute, Villigen PSI, Switzerland.

The original Article has been corrected.

