# Supplementary material for: Intrinsic chiral field as vector potential of the magnetic current in the zig-zag lattice of magnetic dipoles
Source: Sci Rep. 2023 Jan 23;13:1245. doi: 10.1038/s41598-023-28545-9 (PMC9870917; doi:10.1038/s41598-023-28545-9)
Supplement: Supplementary file 1 — Supplementary Information 1. [file 41598_2023_28545_MOESM1_ESM.pdf]

# Supplementary Information

# Intrinsic chiral field as vector potential of the magnetic current in the zig-zag lattice of magnetic dipoles.

Paula Mellado and Andres Concha

*Facultad de Ingenieria y Ciencias, Universidad Adolfo Ibáñez, Santiago, Chile*

Kevin Hofhuis

[a] *Laboratory for Mesoscopic Systems, Department of Materials, ETH Zurich, Switzerland*

[b] *Laboratory for Multiscale Materials Experiments, Paul Scherrer Institute, Switzerland*

[c] *Department of Applied Physics, Yale University, New Haven, USA*

Ignacio Tapia

*Facultad de Ciencias, Departamento de Fisica, Universidad de Chile, Casilla 653, Santiago, Chile*

# I. SUPPLEMENTARY METHODS

## A. Mapping the Dipolar energy to symmetric and chiral Dzyaloshinskii–Moriya exchange.

### 1. Definitions

Sublattice $c$ contains $n_c$ dipoles with magnetization unit vector

$$\hat{m}_k^c = (\sin\theta_k^c, 0, \cos\theta_k^c)$$

and located at positions:

$$\boldsymbol{r}_k^c = (k, 0, 0)\,.$$

Sublattice $p$ contains $n_p = n_c - 1$ dipoles with magnetization unit vector

$$\hat{m}_k^p = (0, \sin\theta_k^p, \cos\theta_k^p)$$

and located at positions:

$$\boldsymbol{r}_k^p = (k, \ell, 0)\,.$$

The length $\ell = y/\Delta$ sets the size of the vertical bond between sublattices c and p and will be a parameter to be tuned in this problem. All dipoles have length $a = 1$ and magnitude of magnetization $m_0$. The angle $\theta_i$ is measured with respect to the $\hat{z}$ axis. $\Delta$ sets the lattice constant along the $\hat{x}$ axis in both chains (distance between the centers of nearest neighbor dipoles located in the same chain).

## B. Energy in terms of vectorial invariants.

The magnetic dipolar energy reads

$$U = \frac{g}{2}\sum_{i\neq k}\frac{1}{|\boldsymbol{r}_i - \boldsymbol{r}_k|^3}\left(\boldsymbol{m}_i\cdot\boldsymbol{m}_k - 3\frac{(\boldsymbol{m}_i\cdot(\boldsymbol{r}_i-\boldsymbol{r}_k))(\boldsymbol{m}_k\cdot(\boldsymbol{r}_i-\boldsymbol{r}_k))}{|\boldsymbol{r}_i-\boldsymbol{r}_k|^5}\right)\,, \tag{1}$$

where $g = \frac{\mu_0 m_0^2}{4\pi\Delta^3}$.

Computing:

$$\boldsymbol{d}_{ik}^{c} = (\boldsymbol{r}_i^c - \boldsymbol{r}_k^c) = (i-k,0,0)\ ,$$

$$\boldsymbol{d}_{ik}^{p} = (\boldsymbol{r}_i^p - \boldsymbol{r}_k^p) = (i-k,0,0)\ ,$$

$$\boldsymbol{d}_{ik}^{cp} = (\boldsymbol{r}_i^c - \boldsymbol{r}_k^p) = \left(i-k+\frac{1}{2},\ell,0\right),$$

$$\hat{m}_i^c \cdot \hat{m}_k^c = \cos\theta_i^c \cos\theta_k^c + \sin\theta_i^c \sin\theta_k^c = \cos(\theta_i^c - \alpha_k^c)\,, \tag{2}$$

$$\hat{m}_i^p \cdot \hat{m}_k^p = \hat{m}_i^c \cdot \hat{m}_k^c, \tag{3}$$

$$\hat{m}_i^c \cdot \hat{m}_k^p = \cos\theta_i^c \cos\theta_k^p, \tag{4}$$

$$\frac{(\boldsymbol{m}_i^c \cdot (\boldsymbol{r}_i^c - \boldsymbol{r}_k^c))(\boldsymbol{m}_k^c \cdot (\boldsymbol{r}_i^c - \boldsymbol{r}_k^c))}{l\boldsymbol{r}_i^c - \boldsymbol{r}_k^c|^5} = \sin\theta_i^c \sin\theta_k^c, \tag{5}$$

$$\frac{(\boldsymbol{m}_i^p \cdot (\boldsymbol{r}_i^p - \boldsymbol{r}_k^p))(\boldsymbol{m}_k^p \cdot (\boldsymbol{r}_i^p - \boldsymbol{r}_k^p))}{|\boldsymbol{r}_i^p - \boldsymbol{r}_k^p|^5} = 0\,, \tag{6}$$

$$\frac{(\boldsymbol{m}_i^c \cdot (\boldsymbol{r}_i^c - \boldsymbol{r}_k^p))(\boldsymbol{m}_k^p \cdot (\boldsymbol{r}_i^c - \boldsymbol{r}_k^p))}{|\boldsymbol{r}_i^c - \boldsymbol{r}_k^p l^5} = \frac{\ell(i-k+\frac{1}{2})}{(i-k+\frac{1}{2})^2+\ell^2}\sin\theta_i^c \sin\theta_k^p, \tag{7}$$

$$(\boldsymbol{m}_i^c \times \boldsymbol{m}_k^c) = (0, -\sin(\theta_i^c - \theta_k^c), 0)\,, \tag{8}$$

$$(\boldsymbol{m}_i^p \times \boldsymbol{m}_k^p) = (\sin(\theta_i^p - \theta_k^p), 0, 0), \tag{9}$$

$$(\boldsymbol{m}_i^c \times \boldsymbol{m}_k^p) = (\cos\theta_i^c \sin\theta_k^p, -\sin\theta_i^c \cos\theta_k^p, \sin\theta_i^c \sin\theta_k^p)\,. \tag{10}$$

$$\tag{11}$$

### 1. *Energy in terms of angles*

The dipolar energy of the system can be written as:

$$U = (U_c + U_p + U_{cp})\,, \tag{12}$$

where $U_c$ is the dipolar interaction between magnets belonging to sublattice c, $U_p$ is the dipolar interaction between magnets belonging to sublattice p and $U_{cp}$ is the interaction between magnets belonging to sublattice c and p. In terms of angular variables these energy contributions become in units of $\frac{g}{2}$:

$$\begin{aligned}
U_c &= \frac{1}{2^3}\sum_{i\neq k}\frac{1}{|i-k|^3}\left[\cos(\theta_i^c - \theta_k^c) - 3\sin\theta_i^c \sin\theta_k^c\right]\ ,\\
U_p &= \frac{1}{2^3}\sum_{i\neq k}\frac{1}{|i-k|^3}\left[\cos(\theta_i^p - \theta_k^p)\right]\ ,\\
U_{cp} &= \sum_{i\neq k}\left[\frac{1}{(\ell^2+(i-k)^2)^{3/2}}\left(\cos\theta_i^c \cos\theta_k^p - 3\frac{\ell(i-k+\frac{1}{2})}{(i-k+\frac{1}{2})^2+\ell^2}\sin\theta_i^c \sin\theta_k^p\right)\right]\ ,
\end{aligned} \tag{13}$$

Now using the identities from the previous section we find:

$$U_c = \frac{1}{8}\sum_{i\neq k}\frac{1}{|i-k|^3}\left[-\frac{1}{2}\hat{m}_i^c\cdot\hat{m}_k^c + \frac{3}{2}\cos(\theta_i^c+\alpha_k^c)\right] ,$$

$$U_p = \frac{1}{8}\sum_{i\neq k}\frac{1}{|i-k|^3}\left[\hat{m}_i^p\cdot\hat{m}_k^p\right] , \tag{14}$$

$$U_{cp} = \sum_{i\neq k}\left[\frac{1}{\left(\ell^2+(i-k+\frac{1}{2})^2\right)^{3/2}}\left(\cos\theta_i^c\cos\theta_k^p - 3\frac{\ell(i-k+\frac{1}{2})}{(i-k+\frac{1}{2})^2+\ell^2}\sin\theta_i^c\sin\theta_k^p\right)\right] , \tag{15}$$

$$U_{cp} = \sum_{i\neq k}\left[\frac{1}{\left(\ell^2+(i-k+\frac{1}{2})^2\right)^{3/2}}\left(\frac{1}{2}\hat{m}_i^c\cdot\hat{m}_k^c - 3\frac{\ell(i-k+\frac{1}{2})}{(i-k+\frac{1}{2})^2+\ell^2}\hat{z}\cdot(\hat{m}_i^c\times\hat{m}_k^p)\right)\right] . \tag{16}$$

And the dipolar energy becomes:

$$U = \sum_{i\neq k}\left[J_{ik}^0\left(-\frac{1}{2}\hat{m}_i^c\cdot\hat{m}_k^c + \frac{3}{2}\cos(\theta_i^c+\theta_k^c) + \hat{m}_i^p\cdot\hat{m}_k^p\right) + J_{i,k}\left(\hat{m}_i^c\cdot\hat{m}_k^p\right) + \boldsymbol{\mathcal{D}}_{ik}\cdot\left(\hat{m}_i^c\times\hat{m}_k^p\right)\right] , \tag{17}$$

where

$$J^0 = \frac{g}{2}\frac{1}{|i-k|^3}$$

and

$$J(\ell) = \frac{g}{2}\frac{1}{\left(\ell^2+(i-k+\frac{1}{2})^2\right)^{3/2}}$$

are respectively the exchange coupling between dipoles belonging to same and different chains. The exchange couplings decay with the distance between dipoles and the distance between chains. The third term in the right hand side of Eq. 17 is a Dzyaloshinskii–Moriya (DM) antisymmetric type of exchange perpendicular to the plane of the system,

$$\boldsymbol{\mathcal{D}}(\ell) = \frac{g}{2}\left(0, 0, -3\frac{\ell(i-k+\frac{1}{2})}{\left((i-k+\frac{1}{2})^2+\ell^2\right)^{\frac{5}{2}}}\right) .$$

### 2. *Energy contributions as a function of interdipolar and interchain distance*

The Dzyaloshinskii–Moriya (DM) coupling between two dipoles at a distance $|(x,y)|$ apart is perpendicular to the plane,

$$\boldsymbol{\mathcal{D}}(x,y) = -3\frac{y(x+\frac{1}{2})}{\left((x+\frac{1}{2})^2+y^2\right)^{\frac{5}{2}}}\hat{z}$$

in units of $\frac{g}{2}$. Fig.S6 (a) shows $\boldsymbol{\mathcal{D}}(x,y)$ in terms of x and y. While in terms of x the decay from zero is monotonic, in terms of y $\boldsymbol{\mathcal{D}}(x,y)$ reaches a maximum for nearest neighbor dipoles $x=0$ at $y=0.25$. Indeed, the maximum of $\boldsymbol{\mathcal{D}}(x,y)$ occurs at the optimum interchain distance $y^O=\frac{1}{4}(1+2x)$. $y^O$ maximizes the DM coupling to

$$\boldsymbol{\mathcal{D}}(x)^{\max}=\frac{384}{25\sqrt{5}\left((2x+1)^2\right)^{3/2}}\hat{z}\,.$$

Fig.S6(c) shows $\boldsymbol{\mathcal{D}}(x)^{\max}$ in terms of x in units of $\frac{g}{2}$, where $x=0$ means nearest neighbors, $x=1$ second nearest neighbors and so on. The figure shows that the DM coupling is relevant for the system up to the second nearest neighbors. One can estimate the effective chiral field that dipoles in chain $p$ exert on dipoles in $c$ by adding out the x component up to third nearest neighbors which yields

$$\boldsymbol{\mathcal{D}}_{\rm ef}=\frac{3\ell}{2\left(\ell^2+\frac{1}{4}\right)^{5/2}}+\frac{9\ell}{2\left(\ell^2+\frac{9}{4}\right)^{5/2}}+\frac{15\ell}{2\left(\ell^2+\frac{25}{4}\right)^{5/2}}$$

which is shown in Fig.S6(b).

As shown in the paper, $J_{ik}$ and $J^0_{ik}$ define respectively the intersublattice symmetric energy $U_{cp}$ and the intra-chain symmetric energy contributions $U_c$ and $U_p$. We can write these coupling in terms of the dipolar distance along x and y direction as $J(x,y)=\frac{1}{\left(\left(x+\frac{1}{2}\right)^2+y^2\right)^{3/2}}$ and $J(x,y)^0=\frac{1}{2\left(\left(x+\frac{1}{2}\right)^2\right)^{3/2}}$. One can estimate the effective symmetric field that dipoles in chain $p$ exert on dipoles in $c$ by adding out the x component up to third nearest neighbors $J_{\rm ef}=2(1/(1+4\ell^2)^{3/2}+1/(9+4\ell^2)^{3/2}+1/(25+4\ell^2)^{3/2})$. The effective couplings $J_{\rm ef}$ and $\boldsymbol{\mathcal{D}}_{\rm ef}$ are compared in supplementary Fig.S7.

### C. Molecular dynamics simulations.

The equation of motion for the polar angle of the inertial magnet located at site $i$ in sublattice $\alpha$, interacting through the full long-range dipolar potential with all other dipoles in the system, reads: $I\frac{d^2\theta_i^\alpha}{dt^2}=\mathcal{T}_i^\alpha-\eta\frac{d\theta_i^\alpha}{dt}$ where $I$ denotes the moment of inertia of the magnets, $\eta$ is the damping for the rotation of dipoles in the lattice[?] and the time $t=y/v=\ell/v$ with v the constant speed of chain $p$. The first term at the right hand side of the previous equation is the intrinsic magnetic torque due to the action of the internal magnetic field due to the dipolar interaction between all dipoles in the system $\mathcal{T}_i^\alpha=\hat{\mathbf{m}}_i^\alpha\times\mathbf{H}_{\rm i}^\alpha$, where $\mathbf{H}_{\rm i}^\alpha=\frac{\partial \rm U}{\partial\hat{\mathbf{m}}_{\rm i}^\alpha}$ denotes the internal magnetic field produced by all dipoles but the *i-th* at the position of $\hat{\mathbf{m}}_i^\alpha$. To solve the previous system of equations, we used a Verlet method. During the simulation interval for the dynamics where sublattice $p$ recedes, the gap increased from $\ell=0$ up to $\ell=\ell_{max}=1.2$. The initial angular positions for the dipoles at $\ell=0$ where $\theta^c=0$ and $\theta^p=\pi$ with a small amount of random disorder added to allow the dynamics and according to experiments. We performed another set of simulations to

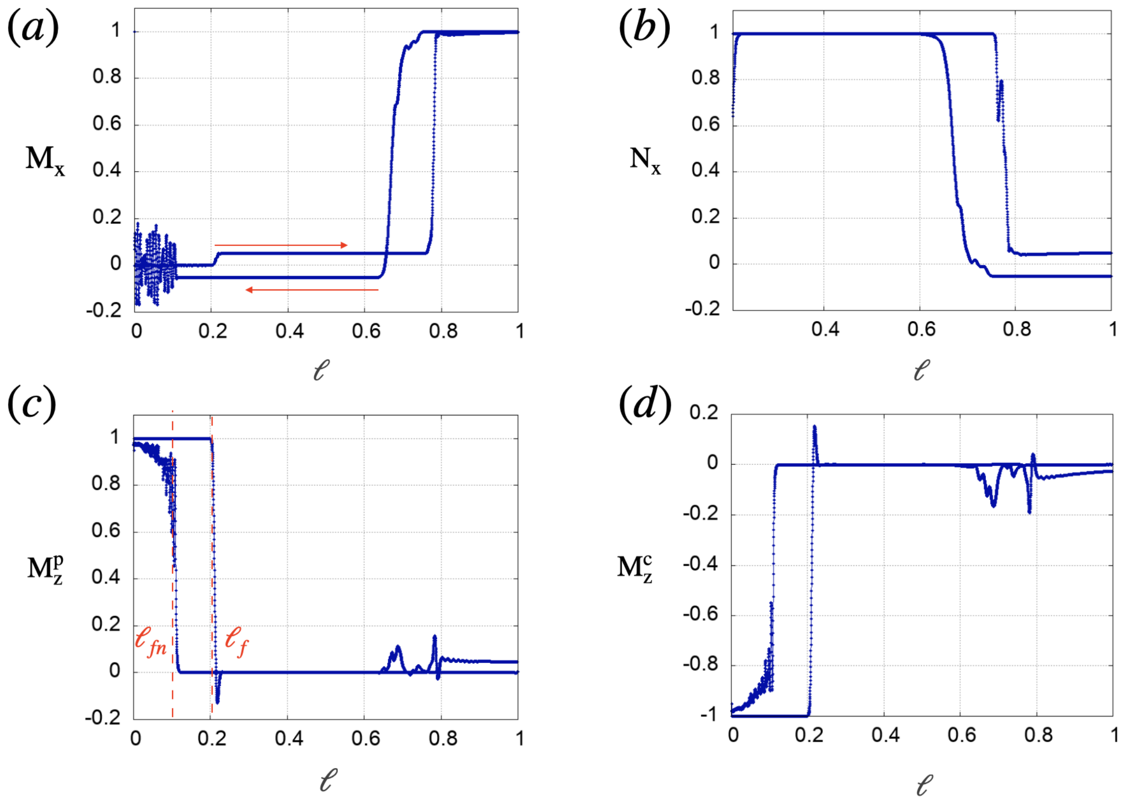


Fig. S1. Magnetization dynamics from molecular dynamics simulations. (a) evolution of $M_x$ and (b) $N_x$ in terms of $\ell$. Red arrows pointing to the right and left denote chain $p$ moving apart from and approaching to chain $c$ respectively. (c) and (d) show respectively the $\hat{z}$ component of the magnetization of the $p$ and $c$ sublattices. An hysteresis loop in the range $\ell \in (\ell_{fn}, \ell_f)$ is apparent showing the metastability of phases $AF^2$ and AF in the numerics.

examine the dynamics when $p$ approaches $c$ with the difference that $\ell$ now is decreased from $\ell_{max}$ back to zero. The system's initial conditions correspond to the final magnetic state of the receding process—the total simulation time corresponded to 2 s in each case. Experimentally measured parameters for lattice constant, damping, inertia, and magnetic charge were used in all simulations. The results from the molecular dynamics simulations were compared with the energy minimization of the system in terms of $\ell$. The numerical minimization of the total energy of the lattice used the 'RandomSearch' method in the numerical minimization routine of Wolfram Mathematica 12.0. The magnets were simulated as mechanical rods with orientation

$$\hat{\mathbf{m}}_i^\alpha = (\cos\phi_i^\alpha \sin\theta_i^\alpha, \sin\phi_i^\alpha \sin\theta_i^\alpha, \cos\theta_i^\alpha)\,, \tag{18}$$

with $(\alpha : \mathrm{c}, \mathrm{p})$ for magnets in the collinear and the parallel chains, respectively. In every simulation we considered $n_c$ magnets in the collinear chain and $n_p = n_c - 1$ in the parallel chain. As the movement of the magnets is constrained to a plane, the angle $\phi_i^\alpha$ is fixed during simulations, and the angle $\theta_i^\alpha$ corresponds to the dynamical variable of the system.

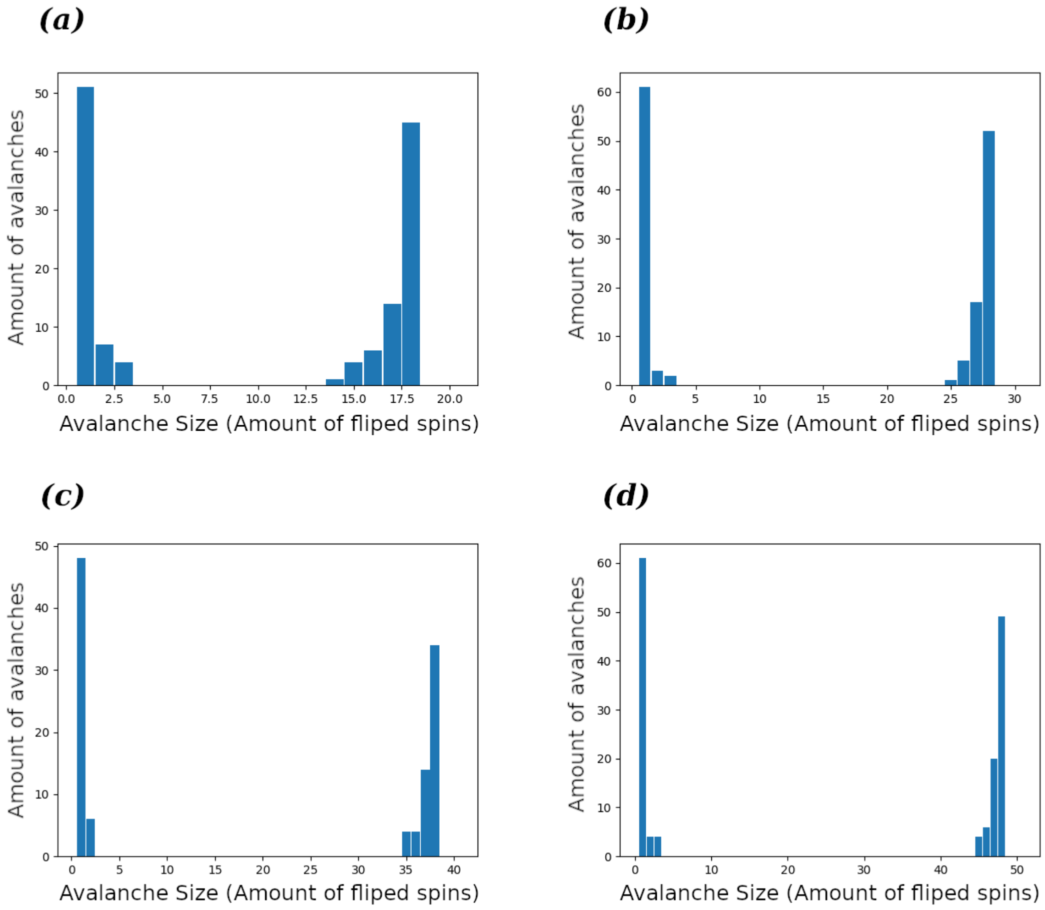


Fig. S2. Histograms for avalanche sizes for 75 simulations with $n = 39$ $(a)$, 75 simulations of size $n = 59$ $(b)$, 56 simulations of size $n = 79$ $(c)$ and 79 simulations of size $n = 99$ $(d)$. A Gaussian noise was inserted in the angle of the plane of rotation with variance 0.01 in order to replicate the geometrical disorder of the experiment.

For magnets in the collinear chain, the plane is fixed at $\phi_i^{\mathrm{c}} = 0$ and at $\phi_i^{\mathrm{p}} = \pi/2$ for magnets in the parallel chain. Aimed to emulate experiments in molecular dynamics we introduced geometrical disorder. This disorder consists in small azimuthal angular variations of the magnets belonging to the two sublattices, which in our experiments have a gaussian distribution with zero mean and standard deviation equal to $\Delta\phi = 0.005$. The evolution equation for $\theta_i^\alpha$ is given by

$$I\ddot{\theta}_i^\alpha = \left( \hat{\mathbf{m}}_i^\alpha \times \sum_{j \neq i} \mathbf{H}_{ij} \right) \cdot \hat{\mathbf{n}}_i^\alpha - \eta(\theta_i^\alpha)\dot{\theta}_i^\alpha \,, \tag{19}$$

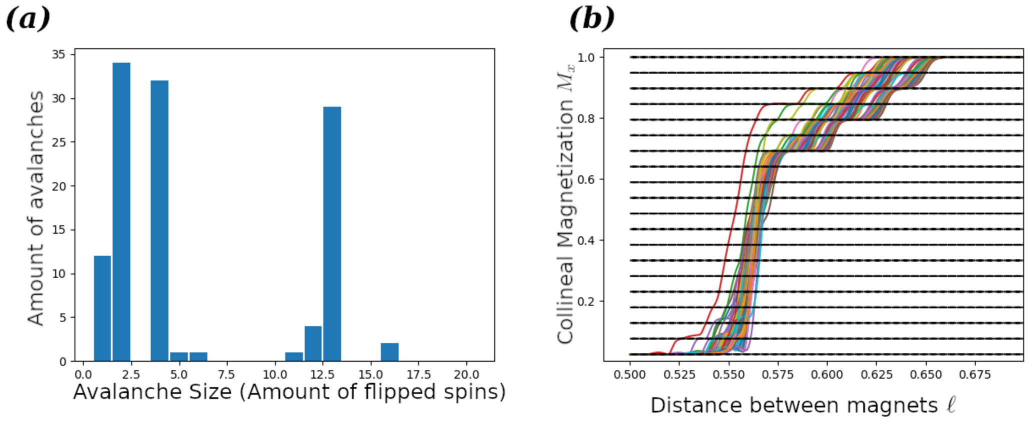


Fig. S3. Results for 72 simulations of $n = 39$ magnets on the collinear chain for the chains approaching from $\ell = 0.5$ to $\ell = 0.7$. $(a)$ shows an histogram of the sizes of the avalanches observed in the 72 simulations. $(b)$ shows the magnetization of the collinear chain around the $x$ axis. Each curve corresponds to a different simulation, while the dashed lines correspond to the values of the magnetization on the different states at which the magnets lie completely at the $x$ axis.

where $\hat{\mathbf{n}}_i^\alpha = (-\sin\phi_i^\alpha, \cos(\phi_i^\alpha), 0)$ is the vector perpendicular to the plane of rotation of the magnet $i$, $\mathbf{H}_{ij}$ is the magnetic field induced by the magnet $j$ over the magnet $i$, given by

$$\mathbf{H}_{ij} = 3\frac{(\mathbf{r}_{ij}\cdot\hat{\mathbf{m}}_i)}{|\mathbf{r}_{ij}|^5}\mathbf{r}_{ij} - \frac{1}{|\mathbf{r}_{ij}|^3}\hat{\mathbf{m}}_i\,, \tag{20}$$

and $\eta$ is a viscosity term given by

$$\eta(\theta) = \frac{500I}{2(\pi/18)^2}\exp\left(-\sin^2(\theta - \frac{\pi}{2})\right) + 10\,. \tag{21}$$

The positions of the magnets are given by $\mathbf{r}_i^{\mathrm{c}} = \Delta(i, 0, 0)$ for the collinear chain and $\mathbf{r}_i^{\mathrm{p}} = \Delta(i, \ell, 0)$ for the parallel chain. The distance between chains is varied by modifying the quantity $\ell$ as the evolution equation is solved. We considered time steps of $\delta t = 2\times 10^{-7}$, at every one of each we moved the chains a distance of $\delta\ell = 1.6\times 10^{-7}$.

We studied the evolution of the magnetization in different regimens. In particular, the $x$ component of the normalized magnetization of the c sublattice is defined as

$$M_x(\ell) = \frac{1}{n_c}\sum_{i=1}^{n_c}\hat{\mathbf{m}}_i^{\mathrm{c}}(\ell)\cdot\hat{\mathbf{x}}\,. \tag{22}$$

Magnetization loops are shown in Fig.S1. At $\ell < \ell_f$ as the two sublattices approach, dipoles in both sublattices struggle to find a stable orientation out of the $x - y$ plane due to the combination of strong interactions and azimuthal

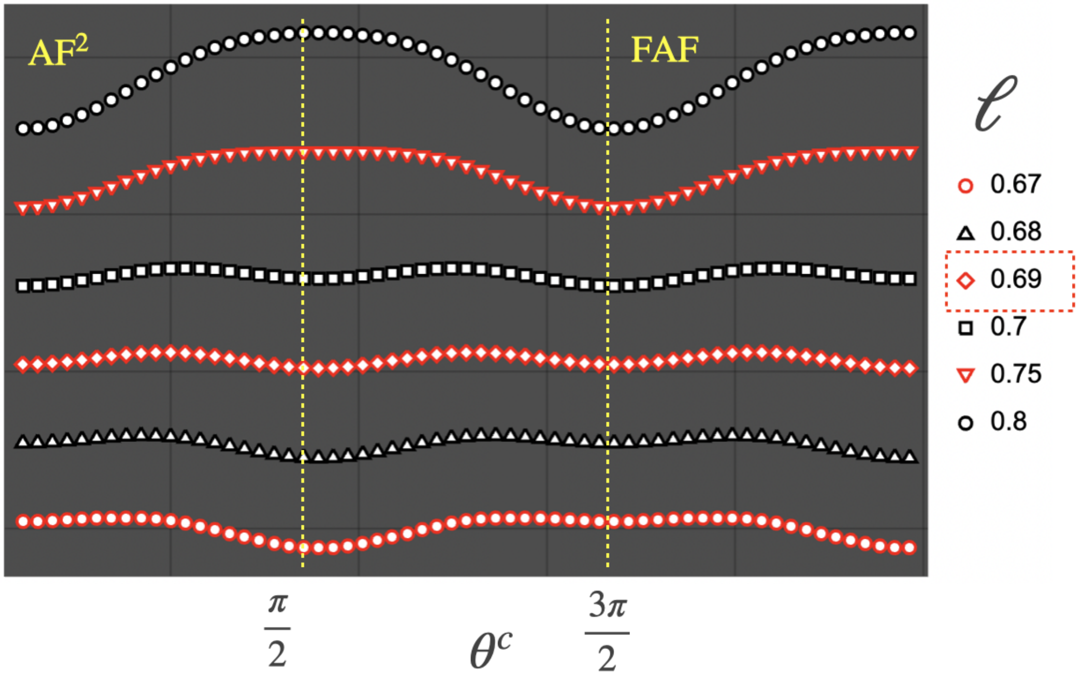


Fig. S4. Energy landscape in terms of the angle of dipoles in sublattice c. Different curves correspond to different values of $\ell$. The curves are displayed vertically for clarity.

disorder. The rapid oscillations of the magnets are registered by the noise of $M_x$ and $M_z^p$ at small $\ell$. The small azimuthal imperfections affect especially dipoles in $p$ due to the rotational symmetry of the antiferromagnetic parallel state. Avalanches could be seen in the transition between FAF and AF$^2$ for both experiments and simulations, as can be seen in Fig.S3(b). A histogram was constructed from 72 simulations where the chains approached each other for different system sizes ($n = 19, 39, 59, 79, 99.$ ). These histograms can be seen in Fig.S2 too. To identify numerically an avalanche we interpolated the magnetization curve for each simulation using cubic splines and then we compute the derivative of the polynomial obtained to search for zeros. These are then filtered to ignore points at which the derivative is zero due to constant magnetization (as at the beginning and end of each simulation) that corresponds to avalanches of size zero. After this filter, we make a histogram of avalanche sizes with data from several simulations.

### 1. *Metastability and Hysteresis at the intermediate regime* $\bar{\ell} < \ell < \ell_*$

The hysteresis loops of Fig.S2 in the manuscript where phases AF$^2$ and FAF are metastable ($\ell \in (\bar{\ell}, \ell_*)$) can be understood from Figs.S4 and S5. In this range of the gap, the dynamics is determined by the magnetization dynamics

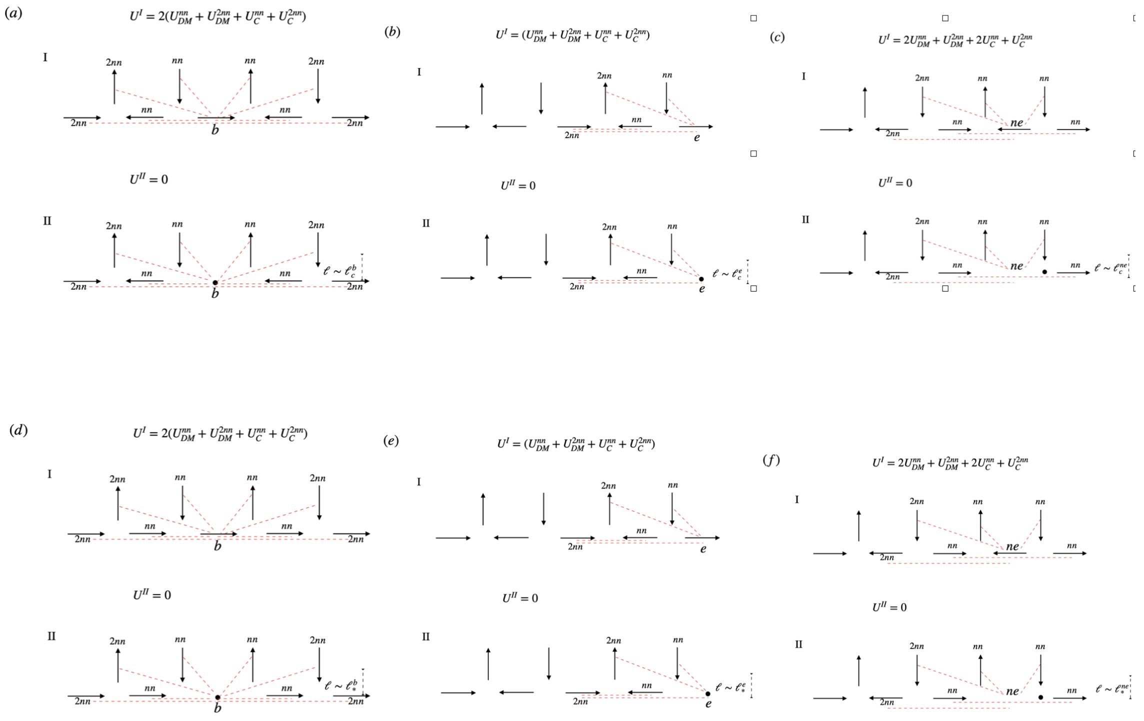


Fig. S5. Energy barriers in a toy model to account for hysteresis.

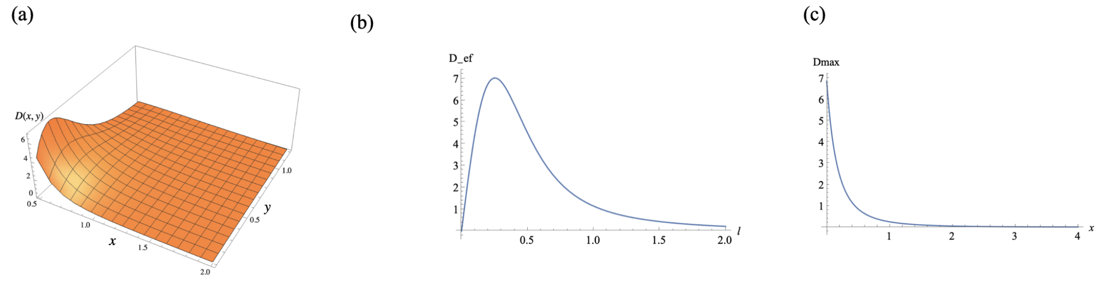


Fig. S6. (a) D coupling (in units of $\frac{g}{2}$) as a function of x and y (b) Effective D coupling, (c) Maximum D coupling.

of sublattice $c$ since $p$ remains in the antiferromagnetic state along $\hat{y}$. Indeed, while the intrachain interactions $U_c$ remains finite and constant, $U_p$ and $U_{cp}$ are negligible and zero respectively while the DM energy decreases with $\ell$ becoming zero at $\ell_c$. The exact formulas for the couplings $J$ and $\mathcal{D}$ reveal a fast decay of interactions with dipolar distance. Consequently, considering interactions up to second nearest neighbors is a good strategy for understanding the behavior of the system in this regime and allows easy estimations of the critical gaps beyond which dipoles in chain $c$ flip. As $\ell$ grows the first dipole to rotate will be the weakest: that is the one that due to its local intrachain magnetic field, needs the smallest magnetic torque from $p$ in order to rotate. Let's consider the case of dipoles at

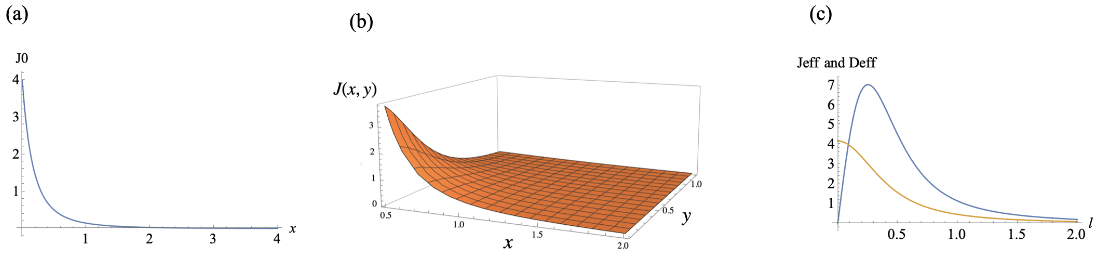


Fig. S7. (a) Symmetric intrasublattice coupling $J^0$, (b) symmetric intersublattice coupling in terms of x and $\ell$ and (c) comparison of effective intersublattice couplings in terms of $\ell$.

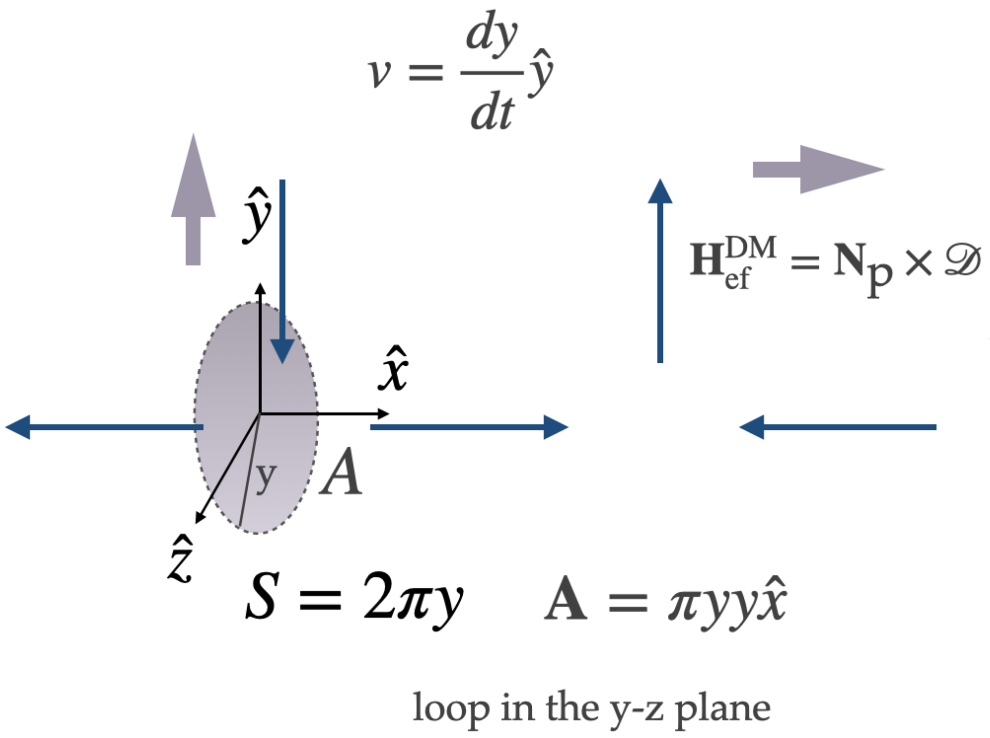


Fig. S8. Illustration to show Faraday'Law in the zig-zag lattice.

the edges, the nearest neighbors of edge dipoles and dipoles in the bulk. Such defect consists of one dipole in $c$ pointing along the $\hat{z}$ axis, which is also known as a Bloch domain wall. Therefore, as $\ell$ grows from $\bar{\ell}$, the chiral field $H^{\mathrm{DM}} = \sum_{i,k} \boldsymbol{m}_k \times \mathcal{D}_{ik}$ acting on dipoles in $c$ must be just sufficient to overcome the energy barrier $\Delta U$ between a pristine magnetic phase (either AF$^2$ or FAF) and the same state with one defect. Indeed, $\Delta U$ is the energy associated with the onset of a Bloch domain wall in the AF$^2$ phase, which in the case of dipoles in the bulk, equals twice the DM energy due to first, *nn* and second nearest neighbors, *2nn*, plus twice the symmetric energy contribution between

nearest neighbors and second nearest neighbors in chain $c$, $\Delta\mathrm{U}^{\mathrm{b}} = 2(\mathrm{U}^{\mathrm{nn}}_{\mathrm{DM}} + \mathrm{U}^{2\mathrm{nn}}_{\mathrm{DM}} + \mathrm{U}^{\mathrm{nn}}_{c} + \mathrm{U}^{2\mathrm{nn}}_{c})$ see Figure S5. For dipoles that are at the edge of $c$ such energy barrier becomes $\Delta\mathrm{U}^{\mathrm{e}} = (\mathrm{U}^{\mathrm{nn}}_{\mathrm{DM}} + \mathrm{U}^{2\mathrm{nn}}_{\mathrm{DM}} + \mathrm{U}^{\mathrm{nn}}_{c} + \mathrm{U}^{2\mathrm{nn}}_{c})$, and for dipoles that are next to edge dipoles $\Delta\mathrm{U}^{\mathrm{ne}} = (2\mathrm{U}^{\mathrm{nn}}_{\mathrm{DM}} + \mathrm{U}^{2\mathrm{nn}}_{\mathrm{DM}} + 2\mathrm{U}^{\mathrm{nn}}_{c} + \mathrm{U}^{2\mathrm{nn}}_{c})$. Which gives respectively $\ell^{b}_{*} = \ell^{e}_{*} \sim 0.7322$ and $\ell^{ne}_{*} \sim 0.7325$, consistent with experimental and numerical results. As the two chains move apart and $\ell \sim 0.7$ all dipoles are candidates to initiate the dynamics. For the approaching dynamics, an analogous analysis yields for the respective critical gaps $\bar{\ell}^{b} = \bar{\ell}^{e} \sim 0.66$ and $\bar{\ell}^{ne} \sim 0.7$. This is consistent with the width of the hysteresis loops $|\bar{\ell} - \ell_{*}| \sim |\bar{\ell}^{b} - \ell^{ne}_{*}|$ in Fig.S2 of the manuscript and shows that the hysteresis in the system is a product of the chiral fields in the zig-zag lattice. Note that in the case when the sublattices approach, there is an accountable difference between the critical fields of dipoles next to the edge, and the rest of the magnets in $c$. Indeed, in this case, dipoles in $c$ connected to one or two hard sites, that is sites connected to two magnetic poles of the same sign, won't fully reverse, because doing so would be energetically very costly. This means that in $c$ the dynamics could begin either at the edge or at the bulk, the final outcome depending on whether or not the weak dipole is connected to a frustrated site. If it is not, all dipoles belonging to that sublattice will eventually reverse, which will define the sense of $\mathrm{M_x}$. The metastability associated with the just described hysteresis is clearly shown in the energy landscape of Figure S4.

### D. Intrinsic magnetic Current and torque

#### 1. *The classical correspondence of the Spin-current*

In? , the spin current arises from the Heisenberg equation whose correspondence to the classical system at hand is:

$$\frac{\partial \boldsymbol{S}_i}{\partial t} = \frac{1}{i\hbar}[\boldsymbol{S}_i, H] = -\sum_k \boldsymbol{\mathcal{J}}_{ki} \,.$$

The correspondence to classical physics is the symplectic evolution:

$$\frac{\partial \boldsymbol{m}_i}{\partial t} = \boldsymbol{m}_i \times \frac{\partial U}{\partial \boldsymbol{m}_i} = \boldsymbol{m}_i \times \boldsymbol{H}_i = \boldsymbol{\mathcal{T}}_i \,,$$

that is

$$\frac{\partial m_i^{(a)}}{\partial t} = \epsilon^{abc} m_i^{(b)} \frac{\partial U}{\partial m_i^{(c)}} \,,$$

here, in $m_i^{(a)}$ the index '$a$' labels the components $x, y, z$ ($\epsilon^{abc}$ is the fully antisymmetric Levi-Civita symbol). Writing down U in terms of $\bar{\mathcal{I}}_{ik}$, the interaction matrix of the system yields,

$$U = -\frac{1}{2}\sum_{i,k} m_i^{(a)} \bar{\mathcal{I}}_{ik}^{(a,b)} m_k^{(b)} \,,$$

one gets

$$\frac{\partial m_i^{(a)}}{\partial t} = -\epsilon^{abc} m_i^{(b)} \sum_k \bar{\mathcal{I}}_{ik}^{(c,d)} m_k^{(d)} = -\sum_k \epsilon^{abc} m_i^{(b)} \bar{\mathcal{I}}_{ik}^{(c,d)} m_k^{(d)} ,$$

thus

$$\mathcal{J}_{ik}^{(a)} = \epsilon^{abc} m_i^{(b)} \bar{\mathcal{I}}_{ik}^{(c,d)} m_k^{(d)} .$$

The matrix elements of the interaction matrix contain the dipolar couplings between all dipoles in the system. It reads:

$$\bar{\mathcal{I}}_{ik} = \begin{pmatrix} \bar{\mathcal{I}}_{i,k}^{(xx)} & 0 & 0 & 0 & \bar{\mathcal{I}}_{i,k}^{(xy)} & 0 \\ 0 & 0 & 0 & \bar{\mathcal{I}}_{ik}^{(yx)} & 0 & 0 \\ 0 & 0 & \bar{\mathcal{I}}_{ik}^{(zz)} & 0 & 0 & \bar{\mathcal{I}}_{ik}^{(zz)} \\ 0 & \bar{\mathcal{I}}_{ki}^{(xy)} & 0 & 0 & 0 & 0 \\ \bar{\mathcal{I}}_{ki}^{(yx)} & 0 & 0 & 0 & \bar{\mathcal{I}}_{ki}^{(yy)} & 0 \\ 0 & 0 & \bar{\mathcal{I}}_{ki}^{(zz)} & 0 & 0 & \bar{\mathcal{I}}_{ki}^{(zz)} \end{pmatrix} ,$$

where

$$\bar{\mathcal{I}}_{ik}^{(ab)} = \frac{\hat{m}_i^{(a)} \hat{m}_k^{(b)} - 3(\hat{m}_i^{(a)} \hat{r}_{ik}^{(a)})(\hat{m}_k^{(b)} \hat{r}_{ik}^{(b)})}{\boldsymbol{r}_{ik}^3} .$$

The matrix elements of $\bar{\mathcal{I}}_{ik}^{(ab)}$ are related to the couplings of Eq.17:

$$\bar{\mathcal{I}}_{ik}^{(xy)} = \mathcal{D}_{ik} ,$$

$$\bar{\mathcal{I}}_{ik}^{(xx)} = -2J_{ik}^0 ,$$

$$\bar{\mathcal{I}}_{ik}^{(yy)} = J_{ik}^0 ,$$

$$\bar{\mathcal{I}}_{ik}^{(zz)} = J_{ik} ,$$

if $i \in \alpha$ and $k \in \beta \neq \alpha$,

$$\bar{\mathcal{I}}_{ik}^{(zz)} = J_{ik}^0 ,$$

if $i \in \alpha$ and $k \in \alpha$. And therefore

$$\frac{\partial \boldsymbol{m}_i}{\partial t} = \boldsymbol{m}_i \times \sum_k \boldsymbol{h}_k = \boldsymbol{m}_i \times \sum_k \left( h_k^{(x)}, h_k^{(y)}, h_k^{(z)} \right) ,$$

with

$$h_k^{(x)} = \left[\bar{\mathcal{I}}_{i,k}^{(xx)} m_k^{(x)} + \bar{\mathcal{I}}_{i,k}^{(xy)} m_k^{(x)}\right] = \left[-2J_{ik}^0 m_k^{(x)} + \mathcal{D}_{ik} m_k^{(x)}\right] ,$$

$$h_k^{(y)} = \left[\bar{\mathcal{I}}_{i,k}^{(xy)} m_k^{(y)} + \bar{\mathcal{I}}_{i,k}^{(yy)} m_k^{(y)}\right] = \left[\mathcal{D}_{ik} m_k^{(y)} + J_{ik}^0 m_k^{(y)}\right] ,$$

$$h_k^{(z)} = \bar{\mathcal{I}}_{i,k}^{(zz)} m_k^{(z)} = (J_{ik} + J_{ik}^0) m_k^{(z)} .$$

*2. Spin current in the x-y phases.*

In phases FAF and $\mathrm{AF}^2$ dipoles lie in the $x-y$ plane, and therefore $\bar{\mathcal{I}}_{i,k}^{(zz)} = 0$ and $m_k^{(z)} = 0$. These forces the spin current to point along $\hat{z}$ and be equal to (in units of g)

$$\boldsymbol{\mathcal{J}}^{(z)} = -2\sum_{i\neq k} m_i^{(x)} \mathcal{D}_{i,k} m_k^{(y)} (\hat{z}) = 2\sum_{i\neq k} m_i^{(x)} \mathcal{D}_{i,k} m_k^{(y)} (\hat{z}) = -2\sum_{i\neq k} \mathcal{D}_{i,k} \sin(\theta_i^\alpha) \sin(\theta_k^\beta)(\hat{z}) .$$

*3. DM vector as a vector potential to the spin current.*

Consider the energy of the system in phase $\mathrm{AF}^2$,

$$U_{\mathrm{AF}^2} = U_c + U_p + U_{DM} = \frac{g}{2} \sum_{i\neq k,\alpha,\beta} \left[\delta^{\alpha\beta} J_{ik}^{\alpha\beta} + (1-\delta^{\alpha\beta})\frac{\mathcal{D}_{ik}}{2}\right] \sin\theta_i^\alpha \sin\theta_k^\beta , \tag{23}$$

where $J_{ik}^{cc} = -2J_{ik}^0$ and $J_{ik}^{pp} = J_{ik}^0$. Now we denote

$$\left[\delta^{\alpha\beta} J_{ik}^{\alpha\beta} + (1-\delta^{\alpha\beta})\frac{\mathcal{D}_{ik}}{2}\right] \equiv \cos A_{ik} \chi_{ik}^{\alpha\beta} . \tag{24}$$

Therefore we get

$$U_{\mathrm{AF}^2} = \frac{g}{2} \sum_{i\neq k,\alpha,\beta} \cos A_{ik} \chi_{ik}^{\alpha\beta} \sin\theta_i^\alpha \sin\theta_k^\beta . \tag{25}$$

On the other hand, the magnetic current in phase $AF^2$ reads:

$$\mathcal{J}^{(z)} = -2\sum_{i\in\mathbf{C}}\sum_{k\in P} \mathcal{D}_{ik} m_i^x m_k^y = -2\sum_{i\neq k,\alpha\neq\beta} \mathcal{D}_{ik} \sin(\theta_i^\alpha - \theta_k^\beta + \frac{\pi}{2}) . \tag{26}$$

And we apply a gauge transformation to it,

$$\mathcal{J}^{(z)} = -2\sum_{i\neq k,\alpha\neq\beta} \sin(\theta_i^\alpha - \theta_k^\beta + \frac{\pi}{2} + A_{ik}) \chi_{ik}^{\alpha\beta} . \tag{27}$$

After expanding the $\sin(\theta_i^\alpha - \theta_k^\beta + \frac{\pi}{2} + A_{ik})$ function and noting that $\cos(\theta_i^\alpha - \theta_k^\beta + \frac{\pi}{2}) = 0$ we recover Eq.26.

### E. Electric Polarization and Magnetoelectric effect.

In Eq. 1 in the manuscript among the contributions to the dipolar energy, we identify the last term $U_{DM} = \boldsymbol{\mathcal{D}}_{ik} \cdot (\hat{m}_i^c \times \hat{m}_k^p)$ as an inter-sublattice DM energy. The DM vector field, $\boldsymbol{\mathcal{D}}_{ik} = -3\frac{\ell(i-k+\frac{1}{2})}{\left((i-k+\frac{1}{2})^2+\ell^2\right)^{\frac{5}{2}}}\hat{z}$ gives rise to the magnetic field $\boldsymbol{H}_i^{DM} = \sum_k \boldsymbol{m}_k \times \boldsymbol{\mathcal{D}}_{ik}$ which acts on dipole i in the $c$ chain and points along the $\hat{x}$ axis when the system is in phase AF$^2$. The DM energy can be written in terms of $\boldsymbol{H}_i^{DM}$ as $U_{DM} = \boldsymbol{H}_i^{DM} \cdot \hat{m}_i^c$.

Consider a large zig-zag lattice where

$$\boldsymbol{\mathcal{D}}_{ik} = -3\frac{\ell(i-k+\frac{1}{2})}{\left((i-k+\frac{1}{2})^2+\ell^2\right)^{\frac{5}{2}}}\hat{z} \rightarrow \boldsymbol{\mathcal{D}}(x,y) = -3\frac{yx}{(x^2+y^2)^{\frac{5}{2}}}\hat{z}\,.$$

We can compute the total effective DM magnetic field that acts on chain $c$ by integrating out $\boldsymbol{\mathcal{D}}(x,y)$ along the $\hat{x}$ direction such that

$$\boldsymbol{H}_{\text{ef}}^{DM} = N_p \int_{1/2}^{\infty} \mathcal{D}(x,y)dx = N_p \frac{8y}{(1+4y^2)^{\frac{3}{2}}}\hat{x}\,,$$

where $N_p$ is the staggered magnetization of chain $p$ along $\hat{y}$.

The effective magnetic field along $\hat{x}$, pierces an area element $\boldsymbol{d\mathcal{A}}$ in the $y-z$ plane and gives rise to a magnetic flux $\varphi^{DM} = \boldsymbol{H}_{\text{ef}}^{DM} \cdot \mathcal{A}$ as illustrated in Figure S8. Because $c$ moves at a constant speed $\boldsymbol{v} = \frac{dy}{dt}\hat{y}$, the magnetic flux changes with time and due to Faraday's law originates a fem

$$\mathcal{E}^{DM} = vzH_{\text{ef}}^{DM}\,.$$

When $\mathcal{E}^{DM}$ is integrated along a loop of perimeter $S = 2\pi z$, it induces an electric field along the $\hat{z}$ axis:

$$\mathcal{E}^{DM} = \oint \boldsymbol{E}^{DM} \cdot dS = E^{DM} 2\pi z = vzH_{\text{ef}}^{DM}\,.$$

And finally the induced electric field equals:

$$\boldsymbol{E}^{DM}(y) = \frac{\boldsymbol{v} \times \boldsymbol{H}_{\text{ef}}^{DM}}{2\pi} = \frac{4yvN_p^s}{(1+4y^2)^{\frac{3}{2}}\,\pi}\hat{z}\,.$$

In a large zig-zag lattice the effective energy in phase $AF^2$ is given by

$$U_{\text{ef}} = U_c^{gs} - g\sum_{i\neq k} J_{ik}^0 \hat{m}_i^c \cdot \hat{m}_k^c - \boldsymbol{H}_{\text{ef}}^{DM} \cdot \hat{m}_i^c = U_c^{gs} - g\sum_{i\neq k} J_{ik}^0 \hat{m}_i^c \cdot \hat{m}_k^c - \frac{2\pi}{v}(\hat{\boldsymbol{m}}_i^c \times \boldsymbol{E}^{DM}) \cdot \hat{y}\,,$$

where $U_c^{gs}$ is the energy of sublattice $c$ in its antiferromagnetic state. This leads to the coupling between the magnetic field and the electric field polarization

$$\mathbf{P} = \frac{\partial U_{AF^2}}{\partial \boldsymbol{E}^{DM}}\,,$$

which is a realization of the linear magnetoelectric effect in this system.

### F. Twisted phase

In dipoles at the c sublattice, azimuthal disorder gives rise to a small transverse component of the magnetization along the $\hat{y}$ direction while for dipoles in p, a small component of the magnetization along the $\hat{x}$ direction arises. Tw phase comprises dipoles in sublattice c in the ferromagnetic collinear state and dipoles in p in an antiferromagnetic twisted chiral configuration. Next we define a wavevector $q_x = \frac{2\theta_j}{j\Lambda} = \frac{\theta(x)}{x}$ and consider the Neel order in $p$ constrained in the $y-z$ plane, $N_p = (0, \sin\theta, \cos\theta)$. The angle $\theta(x,t)$ parametrizes the local magnetic state of p where $t = \ell/v$. Consider the full dipolar energy between two dipoles in the regime $\ell > \ell_*$ in term of $q_x$:

$$U_{i,k} = U_c/n + J^0_{i,k\in p}\cos(\frac{q_x\Delta}{2}(i-k)) + J_{i\in p,k\in c}\sin\left(\frac{q_x\Delta}{2}i\right) + \mathcal{D}_{i\in p,k\in c}\sin\left(\frac{q_x\Delta}{2}i\right)$$

. The full energy in the Tw phase is

$$U = U_c + \sum_{s=|i-k|=1} J^0_s \cos\left(\frac{q_x\Delta}{2}s\right) + \sum_{k=1,k\in c}\sum_{j=1,j\in p}(J_{j,k} + \mathcal{D}_{j,k})\sin\left(\frac{q_x\Delta}{2}j\right) \tag{28}$$

We have computed each energy contribution in Tw phase and found that $U_{DM} = U_{cp} = 0$. Therefore the wavevector $q_x$ minimizes $U_c + U_p$ subject to the constraint $U_{DM} = U_{cp} = 0$. Because of the fast decay of the couplings $\mathcal{D}$ and $J$, we consider interchain interactions up to second nearest neighbor dipoles. Denoting $\omega_1 = (J^0(1) + J(1,\ell) + \mathcal{D}(1,\ell))$ and $\omega_2 = (J(2,\ell) + \mathcal{D}(2,\ell) + \sum_2^n J^0(x))$, the magnetic texture is given by the solution to the equation $\omega_1\sin(\theta) + \omega_2\sin(2\theta) = 0$ which yields

$$\theta(x,t) = 2\arctan \pm\frac{\sqrt{4\omega_2^2 - \omega_1^2}}{\omega_1} \tag{29}$$

that corresponds to a one dimensional soliton. It describes a domain wall with a topological charge $\xi = \pm 1$. A domain wall interpolates between two ground states ($\theta = 0$ or $\pi$), the sign of its topological charge defines its chirality. The net total topological charge of a magnetic strip is a conserved quantity, with the conservation law $\partial_t\rho + \partial_x j = 0$, where $\rho = -\partial_x\theta/\pi$ and $j = \partial_t\theta/\pi$ are the topological charge density and current. This continuity equation determines the dynamic of the winding texture as the gap is tuned.

## II. SUPPLEMENTARY DATA

Availability of Data and Materials: The datasets used and analyzed during the current study are available from the corresponding author upon reasonable request.

## III. SUPPLEMENTARY VIDEO LEYENDS

Video 1. Movie showing the magnetization dynamics of the experimental system when sublattice p is approaching sublattice c. The black dots in the magnets highlight their S pole.

Video 2. Movie showing the magnetization evolution of the dipoles obtained from the molecular dynamics simulations in the case when sublattice p is approaching sublattice c.

Video 3. Movie obtained from the molecular dynamics simulations in the case when sublattice p is receding from sublattice c.
